# Silicic acid limitation drives bloom termination and potential carbon sequestration in an Arctic bloom

**DOI:** 10.1038/s41598-019-44587-4

**Published:** 2019-05-31

**Authors:** Jeffrey W. Krause, Isabelle K. Schulz, Katherine A. Rowe, William Dobbins, Mie H. S. Winding, Mikael K. Sejr, Carlos M. Duarte, Susana Agustí

**Affiliations:** 1 Dauphin Island Sea Lab, Dauphin Island, AL USA; 2 Department of Marine Sciences, University of South Alabama, Mobile, AL USA; 3 Red Sea Research Center, King Abdullah University of Science and Technology, Thuwal, Saudi Arabia; 4 Greenland Climate Research Centre, Greenland Institute of Natural Resources, Nuuk, Greenland; 5 Arctic Research Center (ARC), Aarhus University, Aarhus, Denmark

**Keywords:** Ocean sciences, Biogeochemistry

## Abstract

The spring diatom bloom in the Arctic Ocean accounts for significant annual primary production leading to the most rapid annual drawdown of water-column *p*CO_2_. Late-winter waters in the Atlantic Arctic & Subarctic Provinces (AASP) have lower silicic acid concentrations than nitrate, which suggests diatom blooms may deplete Si before N. Here we test a facet of the hypothesis that silicic acid limitation terminates the spring diatom bloom in the AASP and the sinking of the senescent and dead diatoms helps drive carbon sequestration. During a 6-week study, diatoms bloomed and progressively consumed silicic acid to where it limited their growth. The onset of growth limitation was concurrent with the minimum *p*CO_2_ in the surface waters and increases in both the proportion of dead diatoms and the diatom assemblage sedimentation rate. Data reanalysis within the AASP shows a highly significant and positive correlation between silicic acid and *p*CO_2_ in the surface waters, but no significant relationship with nitrate and *p*CO_2_ was observed unless data were smoothed. Therefore, understanding the future of the AASP spring diatom bloom requires models that explicitly consider changes in silicic acid supply as a driver of this process.

## Introduction

Polar oceans are characterised by strong spring blooms. These blooms are typically dominated by diatoms and, despite being concentrated within a couple of weeks^1^, they account for much of the annual net primary production^2,3^. Therefore, diatom activity can govern the capacity of polar marine ecosystems to take up atmospheric CO_2_ and fuel the food web in both the Southern^4^ and Arctic^5,6^ Oceans.

Diatoms require silicic acid (Si(OH)_4_) to produce their shells made of biogenic silica (bSiO_2_). In late winter, surface water [Si(OH)_4_] is low in the Arctic Ocean, ~5 µM^7,8^, close to an empirical threshold (2 µM) inferred to support diatoms’ niche for dominance of microphytplankton^9^. Late-winter [Si(OH)_4_] has declined across the Atlantic Arctic & Subarctic Provinces (AASP)^8^ in the last three decades. The pre-bloom [Si(OH)_4_] decline has likely affected the maximum potential diatom biomass during the bloom and has been suggested as a driver for the re-distribution of commercial fish species in the subarctic^10^. Yet Si(OH)_4_ uptake data over the broad region is limited^11,12^.

bSiO_2_ provides diatoms ballast, but cells can remain buoyant due to changes in the cellular constituents which are controlled by metabolic processes^13,14^. In Svalbard, Si(OH)_4_ depletion, associated with intense limitation of diatom Si uptake, potentially limited diatom growth rates even when [bSiO_2_] did not exceed [Si(OH)_4_]^12^. During this same campaign, Agustí *et al*.^15^ hypothesized, based on observations that (a) there was a high fraction of dead diatom cells at stations with low [Si(OH)_4_] and (b) higher sinking rates of dead compared to living diatom cells, that regional [Si(OH)_4_] depletion triggers mortality and subsequent sinking of diatoms. A recent analysis of 11 polar-diatom clones demonstrated that while their allometry scales similar to temperate diatoms, their elemental density (element µm^−3^) for C, N, P and Si is 5-50-fold higher^16^. These authors^16^ suggested this inherently high elemental density in polar diatoms may enhance trophic transfer and could be a mechanistic reason why polar systems can sustain exceptional higher-trophic-level biomass despite low annual primary production. Specifically, if the elemental yield (e.g. C, N, P) per polar diatom consumed is high, then polar zooplankton may gain more nutritional value than consuming the same quantity of lower-latitude diatom cells for a similar species and size. Such results also suggest that per cell, polar diatoms have a higher elemental-export potential, relative to their low-latitude counterparts.

Diatom sedimentation plays a large role in driving the biological carbon pump, particularly in polar waters^17^; therefore, resolving the processes controlling diatom sedimentation are critical for understanding oceanic carbon and silicon fluxes^18^. Combining recent results for Arctic diatom physiology^16^, export fate^15^, and limitation by Si(OH)_4_^12^ suggests that the diatom senescence-aggregation export pathway may be important for driving *p*CO_2_ drawdown in the low [Si(OH)_4_] environment of the AASP. Such an observation is consistent with recent mesocosm findings in Norway (~63.5°N)^19^, showing that at low [Si(OH)_4_] diatom export mediated by a diatom senescence and aggregation pathway is favoured over a diatom→copepod→fecal-pellet pathway.

Here we test a facet of the hypothesis that Si(OH)_4_ limitation terminates the spring diatom bloom in the AASP and infer whether subsequent increases in sinking rates of the senescent and dead diatoms helps to drive carbon sequestration. We combine field observations of phytoplankton processes during a 6-week period in the outer-sill region (coastally influenced) of a Western Greenland fjord within the broader AASP; the timing of sampling encompassed the spring 2017 bloom. Laboratory incubations of field populations were used to quantify the degree to which [Si(OH)_4_] limits diatom Si uptake and reduces diatom growth, and to quantify the changes in sinking velocity of dead and living diatoms during the bloom sequence.

We show that depletion of Si(OH)_4_ halted the net accumulation of diatom bSiO_2_, limited diatom productivity, increased the proportion of dead diatom cells, and led to a 4-fold increase in average sinking velocity for the diatom assemblage at the end of the spring bloom. Therefore, this depletion of Si(OH)_4_ appears to be a probable trigger driving the annual signal in biogenic carbon sequestration. The trends observed at our Greenland study site are consistent with broader trends in the AASP from independent multi-national datasets. The timing of the spring bloom development and termination determined during this study is coincident with the timing of the broader regional depletion of Si(OH)_4_, typically with inorganic nitrogen remaining (as either nitrate or ammonium), and the most rapid rate of annual *p*CO_2_ decline in the broader region. Evidence of a pivotal role for silicon supply and diatom removal in driving the spring bloom, and thus ecosystem dynamics and carbon sequestration in the AASP, suggest that on-going^8^ and future^20^ changes in silicon supply will be an important driver of regional ecosystem change.

## Results and Discussion

### Diatom and nutrient dynamics during the spring bloom

**Figure 1:**
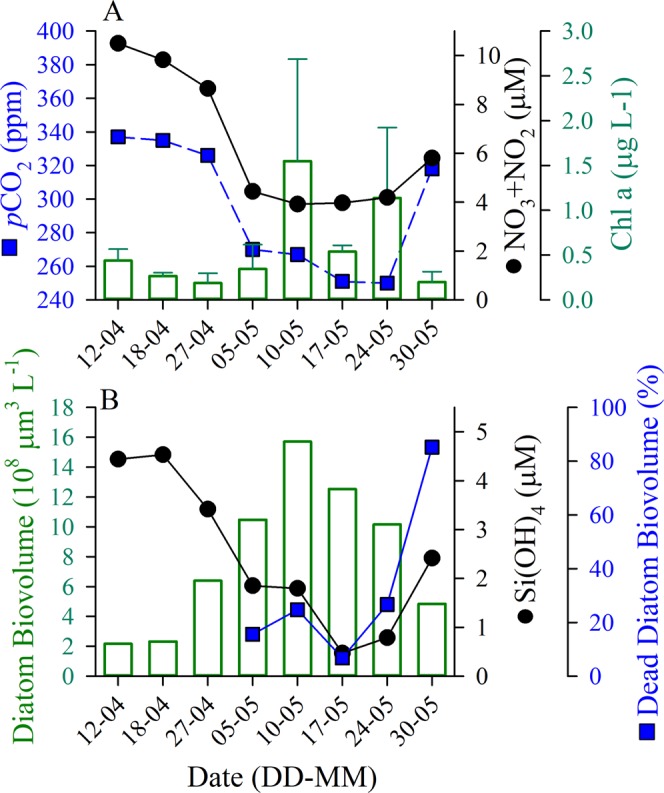
(**A**) Temporal variation during spring 2017 of NO_3_ + NO_2_ (black symbols/line), partial pressure of CO_2_ (*p*CO_2_, blue symbols/line) and Chl *a* (green bars, ± Stdev.) at 5 m depth (station GF3, Godthåbsfjord, Greenland). (**B**) Change in diatom biomass (green bars), Si(OH)_4_ (blue symbols/line) through time at 5-m depth and the percentage of dead diatoms (as biovolume) from 10-m integrated sample through time; diatom viability samples started May 5.

**Figure 2:**
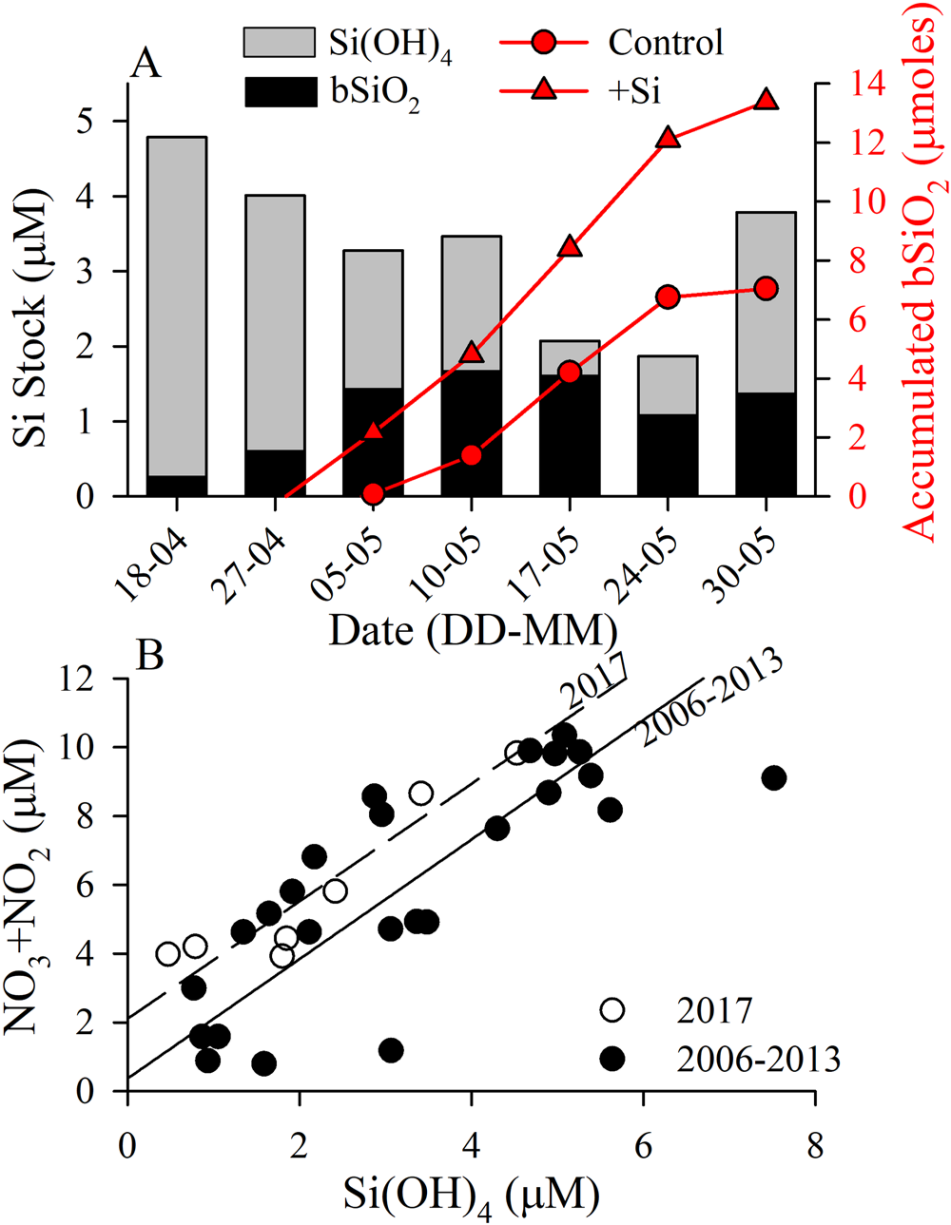
(**A**) Inventory of total Si concentration (bars) distributed between particulate (bSiO_2_, black fill) and dissolved (Si(OH)_4_, grey fill) forms at 5-m depth (station GF3). Superimposed is the total accumulated stock of bSiO_2_, determined from two-day grow-out experimental rates, starting April 18. Rates are both in the control (red circles/line) and +Si (red triangles/line) bioassay treatments. Negative rates observed in late April indicated no net production of bSiO_2_ was quantifiable prior to the onset of the bloom. (**B**) NO_3_ + NO_2_ vs. Si(OH)_4_ for 5-m Niskin samples during 2017 and in previous years from the MarineBasis program at Nuuk (2006–2013). Linear regressions were done using a Model II reduced major axis method.

**Figure 3:**
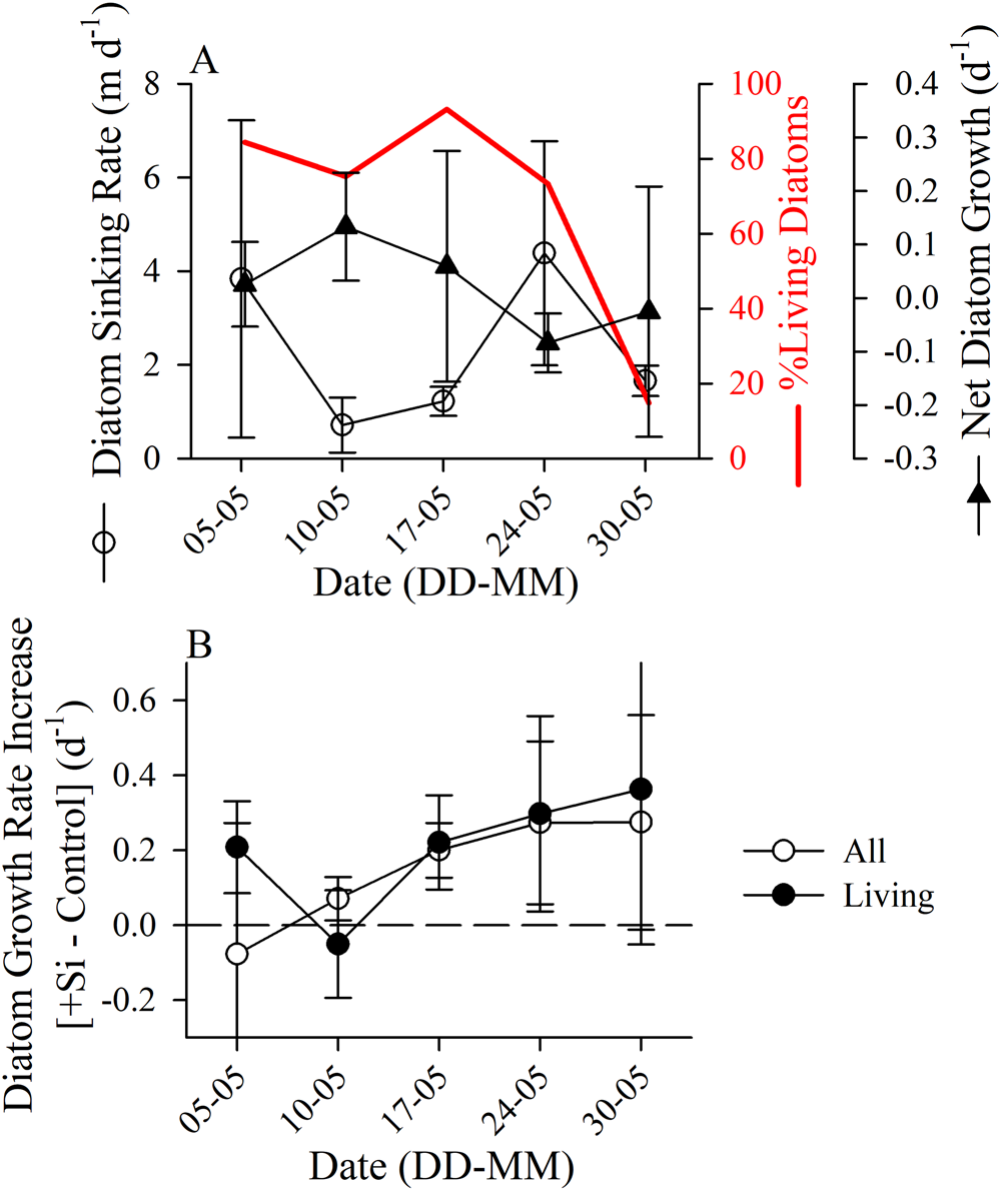
(**A**) Temporal development of diatom net growth rate (filled triangles), sinking rate (open circles), and the percentage of living diatoms (red line) in the last five experiments during May 2017 for material collected in the upper photic zone (surface to 10 m). Error bars are standard deviation. (**B**) Difference in net growth rate for diatoms in the +Si bioassay treatment minus the growth rate in the control, symbols are for living and dead diatoms determined by direct counts of unpreserved material (open circles, denoted as “All”) or living diatoms determined by CDA method (closed circles). Error bars are the 95% confidence interval calculated using a student’s t distribution, which in all cases has a more liberal confidence interval than assuming a normal distribution (i.e. 95% confidence interval range smaller). The zero-difference line is denoted for reference.

Prior to the onset of the spring bloom, phytoplankton biomass was low and inactive (Figs 1, S1), with *p*CO_2_ ~340 ppm. Pre-bloom nutrient concentrations ([Si(OH)_4_] = 5 µM, [NO_3_ + NO_2_] = 10 µM, [PO_4_] = 0.7 µM; Fig. 1) were characteristic of late-winter conditions in the broader region^21,22^. A diatom bloom, dominated by species of *Thalassiosira* and *Bacterosira* (Table S1), initiated by 27 April, whereby chlorophyll *a* (Chl *a*) increased 8-fold to its peak concentration (Fig. 1B). This total phytoplankton biomass change is similar to 6-7-fold increases in two diatom biomass proxies (e.g. biovolume per litre—proxy for biomass which considers both cell size and abundance, [bSiO_2_], Table 1). At the bloom peak, bSiO_2_ accounted for 50–80% of the total Si inventory (Fig. 2A) and *p*CO_2_ declined to a minimum, 250 ppm (17 May, Fig. 1A). As diatom biomass declined (Figs 1B, 2A), [Si(OH)_4_] was ~0.5 µM, while other nutrients were sufficient to maintain primary production among non-diatom phytoplankton ([NO_3_ + NO_2_] = 4 µM; Fig. 1). The first appearance of *Phaeocystis* sp., a flagellate not requiring Si(OH)_4_, was on 17 May, while not directly quantified (both due to the study scope and difficulty in quantifying total abundance because cells may be solitary or in a colony), their presence was more frequently observed in the subsequent weeks (data not shown). The [Si(OH)_4_] depletion was concurrent with increased diatom mortality, as dead diatom cells were 85% of the discernible stock within the photic layer at the end of the bloom (Fig. 3A). By the end of the bloom sequence, 5-m *p*CO_2_ had re-equilibrated with the atmosphere and increased (Fig. 1A).

**Table 1:** Diatom stocks and rates during the 2017 spring bloom cycle (±standard error, n.d. indicates no data).

Day-Month	Si(OH)_4_ (µmol L^−1^)	Diatom Biomass (10^6^ µm^3^ L^−1^)	bSiO_2_ (µmol Si L^−1^)	bSiO_2_ product-ion (µmol Si L^−1^ d^−1^)	bSiO_2_-normalized production (d^−1^)	Net Diatom Growth, (d^−1^)	Weighted Diatom Sedimentation (m d^−1^)	Non-living bSiO_2_ (µmol Si L^−1^)	%bSiO_2_ production met by bSiO_2_ dissolution†
18–04	4.53	233	0.26 ± 0.01	0.00 ± 0.00	0.02 ± 0.01	0.03 ± 0.01	n.d.	0.19*	40.2%
27–04	3.41	639	0.60 ± 0.00	0.01 ± 0.00	0.02 ± 0.00	0.03 ± 0.01	n.d.	0.45*	48.6%
05–05	1.85	1050	1.43 ± 0.12	0.11 ± 0.02	0.07 ± 0.01	0.13 ± 0.05	4.00 ± 3.53	0.27	2.6%
10–05	1.80	1570	1.67 ± 0.03	0.24 ± 0.01	0.13 ± 0.01	0.06 ± 0.02	0.91 ± 0.75	0.65	2.7%
17–05	0.47	1250	1.60 ± 0.11	0.09 ± 0.01	0.05 ± 0.01	−0.08 ± 0.03	1.07 ± 0.27	0.61	6.9%
24–05	0.79	1020	1.08 ± 0.07	0.10 ± 0.00	0.09 ± 0.01	−0.03 ± 0.01	4.29 ± 2.34	0.32	3.1%
30–05	2.41	484	1.37 ± 0.05	0.04 ± 0.02	0.03 ± 0.01	−0.06 ± 0.03	3.81 ± 0.75	0.47	12.9%

Diatom cell counts were converted to biovolume based on morphometrics of each species and summed for biomass measurement. Individual species modal sedimentation rates were determined (Table S2) and the total rate was scaled to diatom contribution to total biovolume (Table S1). Using viability information, i.e. diatom biovolume, the bSiO_2_ pool was split into the fraction associated with non-living diatoms. Based on the non-living-associated bSiO_2_ pool and assuming a specific dissolution rate (†see text) of 0.01 d^−1^, the %bSiO_2_ production supported by bSiO_2_ dissolution (i.e. remineralization) was estimated. *Assumes 75% non-living since no viability information available.

### Rate processes during bloom termination

Diatom activity mirrored temporal trends in stocks. Net bSiO_2_ accumulation and production rates peaked on 5 May, prior to the minimum [Si(OH)_4_] on 17 May (Fig. 2A, Table 1). Polar diatom Si:N is ~1.5^16^, similar to the Si:N drawdown observed 1.7 ± 0.2 (slope in Fig. 2B), suggests that diatoms consumed a majority of the available NO_3_ + NO_2_ during the bloom. The intercept of the relationship, 2.1 ± 0.6 µM [NO_3_ + NO_2_]¸ indicated that if all the Si(OH)_4_ was depleted then sufficient N would be available for non-diatom phytoplankton. The 2017 trend is like previous years (2006–2013) at the time-series site in both the ratio of drawdown, 1.7 ± 0.2, and the positive intercept, i.e. 0.4 ± 0.8 µM [NO_3_ + NO_2_] (Fig. 2B).

[Si(OH)_4_] severely limited diatoms’ production. Si uptake increased linearly with increasing [Si(OH)_4_] (Fig. S2), a trend observed in subtropical gyres^23,24^. At higher [Si(OH)_4_], uptake rates neared 1.0 d^−1^ (Fig. S2), suggesting a decoupling from growth given that net diatom growth rates never approached this magnitude (Fig. 3A), and is consistent with surge Si uptake upon addition of Si(OH)_4_ after short-term Si-starvation^25^. The calculated uptake at the ambient [Si(OH)_4_] (Table 1) was normalized to uptake at 5 µM [Si(OH)_4_] (Fig. S2), the pre-bloom concentration^21^, since uptake did not saturate. 5-µM-normalized Si uptake declined progressively, where ambient [Si(OH)_4_] supported 91% of the 5-µM uptake rate on 18 April (prior to bloom) but <10% between 5–24 May. The lowest percentages coincided with the dates of the highest diatom biomass and [Si(OH)_4_] <2 µM. Si(OH)_4_ limitation of diatom production is also evidenced by the responses to Si additions in bioassay experiments, as the accumulated bSiO_2_ produced over the duration of the bloom would have doubled if diatoms had been supplied an additional 1 µM [Si(OH)_4_] among sampling points (Fig. 2A).

Silicon limitation appeared to limit diatom growth rate. The onset of growth limitation from [Si(OH)_4_] occurred between 10–17 May, as the standing stock of diatom bSiO_2_ exceeded the inventory of Si(OH)_4_ (Fig. 2A). Such yield limitation may be alleviated partially due to rapid bSiO_2_ remineralization, even in ~10 °C upwelling systems^26,27^. However, given the temperature was <1.5 °C (Fig. S1) and the Q_10_ for bSiO_2_ dissolution (2.4, see^28^), the local bSiO_2_ dissolution rates were predicted to be ~0.01 d^−1^ (compared to ~0.05 d^−1^ during upwelling in Monterey Bay, United States^27^). Such a low bSiO_2_ dissolution rate is from direct thermal suppression^29^ and indirect reduction of bacterial activity, a key group for accelerating bSiO_2_ dissolution^30^. Given the bSiO_2_ production rates (Table 1) and high bSiO_2_ standing stock (Fig. 2), most of which was living and not (likely) susceptible to dissolution, bSiO_2_ remineralization would have met between 3–7% of the diatom Si(OH)_4_ demand during the bloom (Table 1). There is also direct evidence for Si limitation of diatom growth. As the bloom progressed, e.g. once [Si(OH)_4_] was <2 µM, the addition of Si made the net diatom growth rate increase significantly (i.e. the 95% confidence interval did not include 0, confirming a positive effect for +Si, Fig. 3B). This direct experimental evidence is not surprising given the intense kinetic limitation (i.e. Si uptake <10% of 5-µM-normalized rate, Fig. S2), which would likely force diatoms to extend the duration of Si uptake during their cell cycle (i.e. slow growth) to meet the minimum amount of Si necessary for their cellular quotas.

Modal diatom sedimentation rates ranged from 0.3 to 8.78 m d^−1^ (slowest and fastest, respectively, resolvable with our experimental approach) across taxa and sampling dates (Table S2). Modal sedimentation rates between living and dead diatoms were not different when examining all data (Mann-Whitney, U = 993, p = 0.40); this was due to significant variability in the sedimentation rates for living (Kruskal-Wallis, H = 32.1, n = 47, p < 0.0001) and dead (Kruskal-Wallis, H = 31.7, n = 47, p < 0.0001) diatoms among sampling dates. The rate of sedimentation for the diatom assemblage (weighted by contribution to biovolume per species) was negatively correlated with the assemblage-average net growth rate for the previous week (i.e. time-lagged correlation: 2-tail Spearman Rho = −0.9, p = 0.037; Table 1). This correlation suggests that as net growth rates increased —driven by healthy, actively-growing populations— the sinking rate of the diatom assemblage decreased. The maximum modal sedimentation rate occurred on 24 May (Fig. 3A), which lagged the lowest [Si(OH)_4_] observed (17 May), consistent with the >3-day lag observed for diatoms persisting in nutrient depleted conditions prior to increased sedimentation^31^ and that the average net diatom growth rate between those time points was negative (17–24 May, Fig. 3A). Hence, we suggest Si(OH)_4_ depletion was the main factor contributing to the increased cell sinking rate.

Prior to Si(OH)_4_ minimum observed on 17 May, multiple proxies demonstrate an active and blooming diatom assemblage including: positive net growth rates for Chl *a* between 26 April to 10 May (Fig. 1A), net accumulation of diatom biomass (Fig. 1B) and bSiO_2_ (Fig. 2A) between 18 April to 10 May, and no significant difference in diatom growth rates between +Si and control treatments in bioassay experiments until 17 May (Fig. 3B). Si(OH)_4_ depletion, despite available [NO_3_ + NO_2_] (~4 µM), corresponded with diatom senescence, presumably turning on programmed cell death pathways^32^. *T. rotula* dominated diatom biomass prior to 24 May; on this same date sedimentation rates increased, and diatoms were declining in both total biomass (Fig. 1B) and the proportion of living cells (Fig. 3A). The observation that Si(OH)_4_ depletion increased cell death rates in a *Thalassiosira*-dominated assemblage has precedence, as high-latitude *Thalassiosira* appear to be more susceptible to increasing cell death rates in response to silicon limitation than other genera, e.g. *Chaetoceros*^33^, perhaps due to *Chaetoceros* escaping the water column prior to Si(OH)_4_ depletion by forming fast-sinking resting spores^34^ (note: *Thalassiosira* spp. also form resting spores but in a previous study^34^ did not do so prior to Si(OH)_4_ depletion). Additionally, Si-limitation in *Coscinodiscus wailesii* (another centric diatom) increases transparent exopolymer particles^35^, which are known to enhance aggregation and export^36^. It has also been proposed that for larger diatoms, post-mortem cell lysis may play a larger role in aggregation^35^. These prior observations are consistent with the results observed in our study where sinking rates increased concurrently with the increase in the proportion of dead diatom cells (Fig. 3A). And beyond marine systems, limnological studies have long suggested that when Si(OH)_4_ is depleted, but other limiting factors are replete, mass diatom mortality can occur^37^.

**Figure 4:**
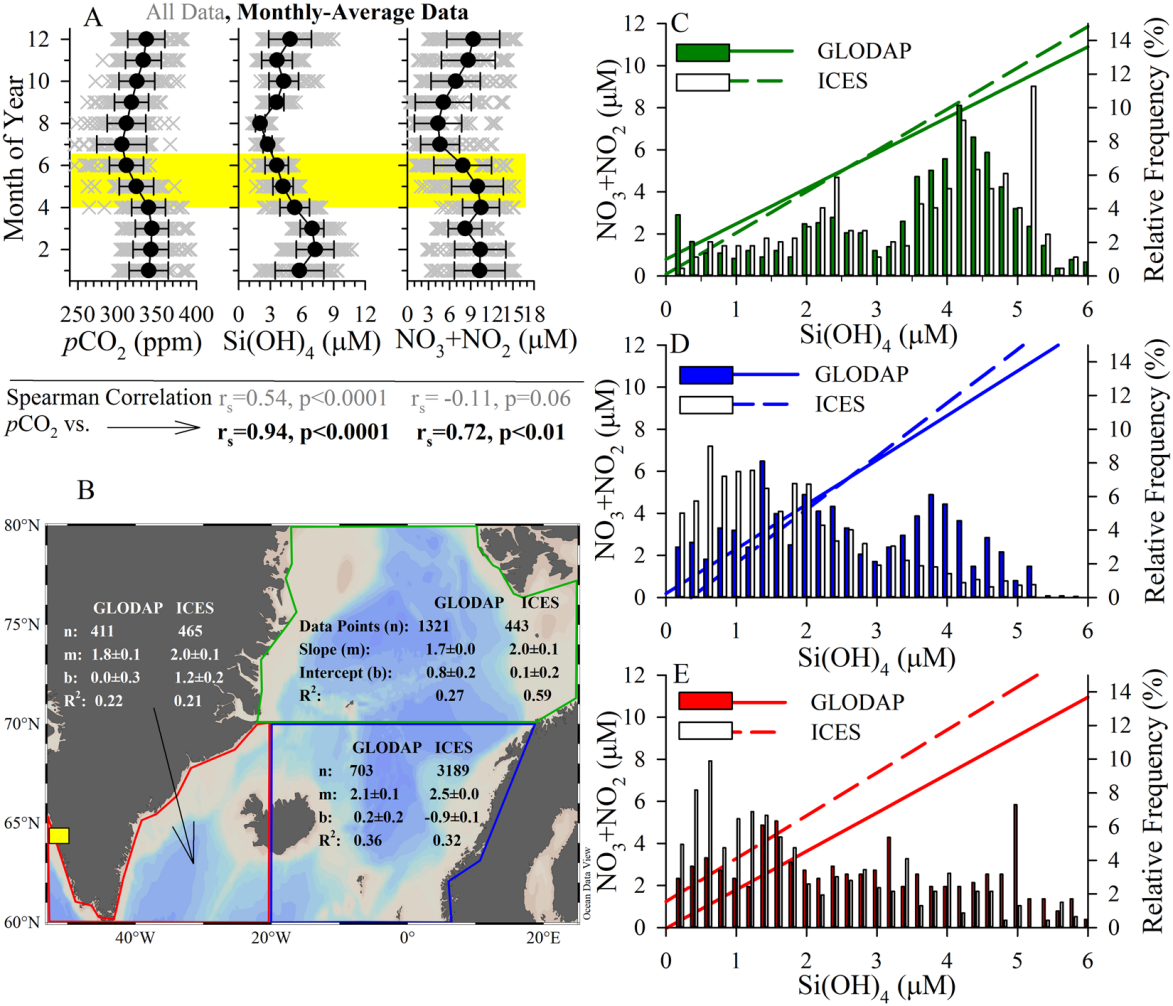
Trends in the broader AASP. (**A**) Gridded *p*CO_2_ (left panel), Si(OH)_4_ (middle panel), and NO_3_ + NO_2_ (right panel) using data from^41^. Grey “X” values are raw data, black filled circles are monthly means (±Stdev.). Below plots are 2-tail Spearman Rho correlation statistics shown for comparison of *p*CO_2_ with each nutrient (statistics below each panel); gravy text is for raw data, black/bold text is for monthly-averaged data. The yellow highlighted area denotes the period of the year examined in all other panels (B–E) with independent data sets. (**B**) Map denoting zonation, database (GLODAP, ICES) metadata and Model II regression statistics (data points “n”, slope “m”, intercept “b”, R^2^) for regressions in panels C–E; MarineBasis station included for reference (yellow square). (**C**–**E**) Regressions for NO_3_ + NO_2_ vs. Si(OH)_4_ for the GLODAP (solid line) and ICES (dashed line) data and a histogram of [Si(OH)_4_] (relative frequency on right y-axis) for the GLODAP (filled bars) and ICES (open bars) data sets in the (**C**) Greenland and Norwegian Seas (Green), (**D**) north-eastern Atlantic (Blue), and (**E**) south-eastern coastal Greenland (Red). Regions were broadly separated by latitude (i.e. 60°–70°N, 70°–80° N), and the lower region separated loosely to reflect the different Longhurst provinces (i.e. combined AASP) associated with waters east and west of Iceland. Individual points colour coded for year of observation, among all domains in both data sets, are shown elsewhere (Fig. S3). For both data sets (**C**–**E**), sample data was confined to the map domain, within the upper 50 m of the water column, and only from April through mid-June.

The results obtained support the hypothesis that Si(OH)_4_ limitation terminates the diatom bloom in the AASP. This is similar to results reported previously^38^, which inferred that increased Si(OH)_4_ supply can prolong a diatom bloom in the southern domain of the AASP. However, this prior study^38^ did not directly test whether diatom growth was limited by Si availability (i.e. only making inferences based on ecological niches^9^ from earlier mesocosm results) and provided no mechanistic explanation regarding the connection between diatom bloom termination and mass export. During our study, net diatom growth effectively stopped when [Si(OH)_4_] was <1 µM. Sedimentation experiments showed, consistent with earlier work using diatom cultures^13,14,39^ and recently in Svalbard^15^, that actively growing diatom cells are able to up-regulate buoyancy while senescent or dead cells sink at faster rates. We observed an inverse relationship between bSiO_2_-normalized production (Table 1), an independent metric of diatom growth from cell counts, and sedimentation rates (Fig. 3A); therefore, Si(OH)_4_ limitation led to diatom mortality and rapid sinking. Si(OH)_4_-limited diatoms in culture have significantly more organic matter than diatoms limited by nitrogen^40^ and polar diatoms have substantially higher elemental density than lower latitude diatoms^16^. Over an annual cycle in the AASP, [Si(OH)_4_] and *p*CO_2_ have a stronger correlation than *p*CO_2_ to [NO_3_ + NO_2_] (Fig. 4A, data from^41^). We suggest that Si(OH)_4_ uptake and depletion regulate the spring diatom bloom, its subsequent collapse, and thus is a main driver of the major spring carbon export event and surface *p*CO_2_ undersaturation —characteristic of the AASP at the end of the spring bloom^42^.

### The role of Silicon in controlling the spring bloom in the Atlantic Arctic & Subarctic Provinces

The results reported are from a coastally-influenced station in a Greenland fjord but are consistent with observations for the broader AASP beyond the highlighted Svalbard studies^12,15^. Paired [Si(OH)_4_] and [NO_3_ + NO_2_] data from a 3.7 × 10^6^ km^2^ subarea in the broader AASP (GLODAPv2 ~2400 observations; ICES Oceanography collections, ~4100 observations), demonstrate the condition of excess N, relative to Si, at the beginning of the season selects for diatom blooms to be limited by Si(OH)_4_ (Fig. 4B–E). Indeed, the intercept of the consumption curves among subregions and data sets range from −0.9 µM [NO_3_ + NO_2_] (i.e. N consumed before Si) to 1.2 µM [NO_3_ + NO_2_] (i.e. Si consumed before N, Fig. 4C–E). The correlation between gridded Si(OH)_4_ and *p*CO_2_ data^41^ is positive and highly significant (2-tail Spearman Rho = 0.54, n = 864, p < 0.0001, Fig. 4A), but the corresponding NO_3_ + NO_2_ correlation with *p*CO_2_ is negative and not significant (2-tail Spearman Rho = −0.11, n = 864, p = 0.06). Only when NO_3_ + NO_2_ are averaged among months and compared to average monthly *p*CO_2_ does the correlation become positive and significant (2-tail Spearman Rho = 0.72, n = 12, p < 0.01), but this monthly averaging also improves the correlation between Si(OH)_4_ and *p*CO_2_ (2-tail Spearman Rho = 0.94, n = 12, p < 0.0001).

Our data suggest that when [Si(OH)_4_] <1 µM, diatom growth limitation occurs from both extreme kinetic limitation for Si uptake and yield limitation due to the high diatom standing stock. If Si(OH)_4_ is growth limiting at ~1 µM, there would be sufficient [NO_3_ + NO_2_] in all data trends from the GLODAP and ICES data sets (Fig. 4C–E) to support further non-diatom primary production. Histograms demonstrate the occurrence of ≤1 µM [Si(OH)_4_] are frequent; thus, the regression slopes and intercepts are not driven by extrapolation or few data points. Similar trends in the relationship between [Si(OH)_4_] and [NO_3_ + NO_2_] have also been observed at higher latitudes (75°–85°N) in the AASP during the GEOTRACES GIPY11 cruise (July 2007; 2.9 ± 0.2 µM NO_3_ + NO_2_ remaining when Si(OH)_4_ depleted); this timing is consistent with the northward progression of the spring bloom in the AASP. In the southern region of our reanalysis domain in the AASP (Fig. 4B), a previous study observed mass export of diatoms due to formation of resting *Chaetoceros* spores, where the trigger for spore formation was coincident with a rapid drawdown of [Si(OH)_4_] (i.e. not full depletion), while [NO_3_ + NO_2_] was replete^34^. Thus, the first pulse of diatom carbon export can start prior to the maximum [Si(OH)_4_] depletion, i.e. prior to the onset of potentially growth limiting concentrations. This demonstrates diatom and Si(OH)_4_ coupling provides multiple modes of high export potential during spring bloom within the broader domain considered due to both growth limitation and settling (e.g. *Thalassiosira* and *Bacterosira*-dominated bloom here) or rapid [Si(OH)_4_] decline, prior to exhaustion, potentially triggering settling (e.g. *Chaetoceros*-dominated assemblage).

Collectively, these three independent data sets suggest that Si(OH)_4_ control of the spring diatom bloom is prevalent across the AASP and is consistent with some model results^43^. Other types of limitation for diatoms within the reanalysis data set do not appear to be as persistent. For instance, iron limitation (as observed in the Southern Ocean) would not be expected to promote full [NO_3_ + NO_2_] drawdown post bloom (Fig. 4A) and fuel the typically observed succession from diatoms to *Phaeocystis* and/or *Micromonas* spp. regionally^12,15^. Additionally, iron limitation increases diatom Si uptake relative to N^44^ and polar diatoms have higher Si:N ratios than lower-latitude diatoms^16^; thus, if iron limitation were the dominant factor regulating diatoms during the spring bloom we would anticipate N:Si depletion slopes (Fig. 4B–E) to be <1 instead of nearly 2.0 (i.e. N depleted twice as fast as Si).

These independent data represent a compilation by scientists from over 10 countries across a broad spatial domain (3.7 × 10^6^ km^2^) spanning over 30 years. The low [Si(OH)_4_] condition in this region during late spring is a robust interannual trend, consistent with our dataset, and suggestive of a spatially-widespread condition when diatoms (vs. *Phaeocystis*) are favored to dominate the spring bloom. Clearly, under conditions when *Phaeocystis* are favored over diatoms, nitrate instead of Si(OH)_4_ will likely be depleted first. However, the annual Si(OH)_4_ drawdown in the GLODAP and ICES data sets corresponds well with the independent *p*CO_2_ data^41^ —where the strong positive correlation between *p*CO_2_ and Si(OH)_4_ is observed— further supporting the proposed link between diatom Si(OH)_4_ limitation and mass carbon export.

Silicon limitation may become more acute in the AASP. Atlantic diatoms within the 50–65°N latitude band are stimulated by increases in nutrient supply from turbulent conditions, e.g. positive phase of the North Atlantic Oscillation^45^. The reported decline in pre-bloom [Si(OH)_4_] since the early 1990s is attributed to decreased winter convection and retraction of the subpolar gyre^8^, and mirrors broader climatological trends reflected by a decline in the Atlantic meridional overturning circulation index during the same period^46^. On top of these basin-scale changes, multiannual sea-ice loss is likely to dilute Si(OH)_4_ pools, as concentrations are already limiting for ice diatoms^47,48^. Both processes, reduced physical convection and sea-ice melt, are predicted to restrict Si supply. In contrast, terrigenous freshwater discharge, with [Si(OH)_4_] ranging from 10–500 µM in glacial rivers, is a significant source —contributing 20 Gmol Si year^−1^ to Greenland coastal waters^20^. Indeed, increased discharge from melting of the Greenland glaciers is predicted to increase Si(OH)_4_ inputs into the Greenland coastal waters 20–160% by 2100^20^. Assuming the current melt was confined to three months and flowed uniformly onto a 1.1 × 10^6^ km^2^ Greenland shelf into a 5-m water column, this would input 0.04 µmol Si L^−1^ d^−1^ (Table S3). Such a Si-delivery rate corresponds represents 16–108% of our measured bSiO_2_ production rates (Table 1). Likewise, [Si(OH)_4_] reaches high levels in Siberian rivers (e.g. ≤160 µM^49^). Whereas sea-ice melting and reduced winter convection may make Si(OH)_4_ limitation more acute in the open AASP, increased discharge from the Greenland Ice Sheet and major rivers discharging may enhance the scope for diatom production later in the season at the land-sea interface if sufficient NO_3_ + NO_2_ is not consumed earlier. Understanding how diatom production will respond to such changes has broad implications for carbon sequestration and fisheries production in Greenland and the broad AASP coastal waters.

Rapid changes in the Arctic are driving efforts to model the likely future of the Arctic Ocean^43^. Whereas these models consider changes in ice loss, freshening, warming, nitrogen and turbidity with climate change, few include Si(OH)_4_ as a possible nutrient limiting diatom growth. Those models including Si suggest that spatially and temporally extensive Si(OH)_4_ limitation in some regions shifts the phytoplankton away from diatoms toward smaller flagellates^43^. Other AASP models which include Si-cycling processes^50,51^ lacked field data to ground truth kinetic parameters. These models used half-saturation constants for Si uptake which are 4-10-fold lower than recent field measurements^12^, and therefore wrongly implied that Si(OH)_4_ does not play an important role for regional diatoms, likely due to artificially enhancing diatoms’ efficiency for Si uptake. Whereas N likely sets the limit to total annual primary production, spring diatom productivity, which plays a significant role in net ecosystem production and driving carbon sequestration^2,52^, appears to be strongly controlled by Si supply. Understanding the future of the spring diatom bloom requires models that explicitly consider changes in Si supply as a driver of this key process.

## Materials and Methods

### Study site and timing

From 12 April to 30 May 2017, eight (~weekly) samplings were performed at station GF3 (64°07′N, 51°53′W, Fig. S4, station location also denoted in Fig. 4B) near the entrance to Godthåbsfjord. The Godthåbsfjord covers 2013 km^2^ (average depth of ∼250 m) with several sills located near the fjord entrance^21^. The sampling station is part of MarineBasis-Nuuk monitoring programme and located in the outer part of the fjord (350 m depth).

### Collection and experimental design

Vertical hydrography, fluorescence and irradiance profiles between were obtained using a SeaBird 19+ CTD profiler (Sea-Bird Scientific, Washington, United States) equipped with a Seapoint (Seapoint Sensors, Inc., New Hampshire, United States) Chl *a* fluorometer, and Biospherical radiometer (Biospherical Instruments, Inc., California, United States), respectively. Manual sampling at 5-m depth was done using 5-L Niskin bottles secured to a line and tripped using a messenger. From the Niskin bottles, water samples were collected for inorganic nutrients, Chl *a*, bSiO_2_, diatom abundance/taxonomy and viability, and Si rate measurements (discussed below).

Two types of 48-hr laboratory experiments were conducted: a bioassay and sedimentation experiment. For the bioassay experiments, four 5-L Niskin bottles were collected, homogenized and drained into two transparent 10-L polyethylene bags. One bag was a control and the other received an addition of sodium metasilicate to increase the [Si(OH)_4_] by 1 μM. Samples were incubated at 1 °C in a cold room illuminated with continuous irradiance (20–70 μE m^−2^ s^−1^) for 48 hours, then sub-sampled for nutrients, bSiO_2_, diatom abundance, and water for additional bSiO_2_ production measurements (see below). For sedimentation experiments, material was collected in the euphotic zone by two vertical net tows in succession using a 15 µm net (Surface to 10 m depth interval). Additional water was collected at 5 m, using Niskin bottles, for these experiments and stored in the dark and ambient temperature until the experiment was set up. Samples were incubated at 1 °C in a cold room but covered with black cloth to force diatoms into darkness (details below).

### Nutrient, particulate, diatom quantification and viability analyses

Samples were analysed using established methods. For nutrients, water was filtered (nominal pore size 0.7 µm), stored in acid-washed 30-mL plastic vials, and frozen until analysis (−18 °C). Samples for inorganic nutrients (nitrate + nitrite, orthophosphate and silicic acid, detection limits 0.10, 0.06 and 0.20 μM, respectively) where measured with flow injection on a Skalar autoanalyzer^52^. Triplicate samples for Chl *a* determination were filtered onto glass-fiber filters (≤0.3 bar), extracted immediately into 10 ml 96% ethanol for 24 hours, then quantified on a fluorometer (TD-700, Turner Designs, CA, USA) calibrated against a pure Chl *a* standard. Partial pressure of CO_2_ was measured at 5 m depth using an infrared CO_2_ analyser (EGM-4, PP Systems as described elsewhere^53^). Triplicate samples (630 mL) for particulate bSiO_2_ were filtered onto a 1.2 μm pore polycarbonate filter and dried at 60 °C in a cryogenic vial, sealed and stored. bSiO_2_ was quantified using a 0.2 normality NaOH digestion in Teflon tubes according to previous methods^54^. Lithogenic silica was quantified, but even assuming a liberal 15% dissolution^55^, did not significantly change the major bSiO_2_ results. Consistent with previous studies, we report bSiO_2_ without lithogenic correction (but corrected data available in Table S4).

The diatom community was analysed using an inverted microscope with a Sedgewick Rafter chamber; this was done for the 5-m Niskin bottle samples and a subsample of the vertical net material used for the sinking-rate experiments starting on 5 May. Subsamples for diatom counts were split into two equal volumes to quantify viability. On one split duplicated total cell counts (dead + live cells) were quantified as described above; no fixatives were used, and samples were counted immediately upon arrival back on shore (e.g. finished within ~1 hour elapsed time from return). Diatom dimensions among species were quantified, to calculate biovolumes, thereby allowing cell concentration per sample volume to be converted to summed diatom biovolume per sample volume allowing for a proxy of biomass. Diatom biovolume and elemental content (e.g. C, N, P, Si) all have been demonstrated to scale together (reviewed elsewhere^16^). For the second split, a cell digestion assay (CDA)^56^ was conducted to quantify abundance of living cells using a method optimized for work with polar phytoplankton^56^. Briefly, for Arctic communities, the CDA involves incubation of fresh samples at 25 °C with an enzymatic cocktail for the digestion of dead cells, i.e. those with compromised membranes. After digestion, abundances were quantified as described above (i.e. for live cells) and the dead cells were calculated by difference from the total. For total (live + dead cells —i.e. what would be quantified without the CDA) and live diatoms (determined by the CDA), the net growth rate was calculated for each species between sampling points assuming logistic growth. An aggregate net-growth rate was determined by averaging all individual species/group growth rates for each sampling point and deriving the net change between sampling points based on a logistic-growth assumption.

Diatom viability data enabled estimating the fraction of bSiO_2_ associated with each pool. Given the total bSiO_2_, we partitioned this based on the proportion of diatom biovolume which was associated with living and dead diatoms. This approach assumes a negligible effect on bSiO_2_ from other phytoplankton (e.g. silicoflagellates), Rhizaria (e.g. polycystine radiolaria, pheaodaria), or diatom detrital fragments. Silicoflagellates and Rhizaria were not present in microscopy samples. During the bloom initiation and building, this assumption was likely valid; however, as the bloom declined and the proportion of dead diatoms increased, there was likely increased detrital fragments present.

### Biogenic silica production and kinetics

Two different experiments were conducted to assess the impact of silicon availability on diatoms. At the time of collection, the kinetics of Si(OH)_4_ uptake were quantified by taking ten subsamples (300 mL per sample), amending seven bottles with sodium metasilicate (three received no addition) to create a concentration gradient between ambient and +20 μM Si(OH)_4_^54^. After 48-hrs in the bioassay experiment, triplicate subsamples were drawn from both the control and +Si container, then prepared for measurement of bSiO_2_ production. To all samples, 260 Bq of high specific activity ^32^Si(OH)_4_ (>20 kBq µmol Si^−1^) was added to each sample and incubated at 1 °C in a cold room illuminated with continuous irradiance (20–70 μE m^−2^ s^−1^) for 24 hours. Samples were filtered using a 1.2-μm pore size polycarbonate filter, dried in a plastic liquid scintillation vial, and aged into secular equilibrium with ^32^P, the daughter isotope of ^32^Si, for four months prior to quantification of sample activity using a liquid scintillation method^54^; the activity signal relative to the blank was typically a factor of 3–7 for the lowest activity samples but this increased by one or two orders of magnitude for high-activity samples (i.e. signal to noise >100). bSiO_2_-normalized rates assume logistic growth^54^. The net bSiO_2_ production rate was also quantified by measuring the net change in bSiO_2_ over 48 hours, with and without a 1-μM [Si(OH)_4_] amendment, compared to the bSiO_2_ at time of sample collection. The standard deviation (average and median coefficient of variation among all triplicates was 11% and 7%, respectively) for bSiO_2_ at each time point was propagated as done previously^54^.

### Estimation of sinking rate and modal-distribution modeling

To measure diatom sinking rates during the 48-hour sedimentation experiment, we used two 1-m height, 0.1-m diameter cylindrical chambers (filled to ~5.0 L) fit with a spigot at the base for sampling. Five litres of the 5-m Niskin sample water was 5-μm filtered and added to each sedimentation chamber. Phytoplankton, which was concentrated with the 15–µm vertical net tows (0–10 m), was gently added at the top of the chamber. An initial subsample was taken to quantify the live vs. dead fraction. Sedimentation chambers were kept in darkness and 200 mL of water was subsampled through the spigot at each time point (2, 6, 10–16, 24 and 48 h). The subsample was concentrated by inverted filtration to a final volume of 10 mL, this volume was then split to quantify total diatoms and living diatoms as discussed above.

Through time, sedimentation rates were determined using a modal-distribution approach, i.e. a conservative approach which is based on observation of quantifying the time point when the highest proportion of cells had settled the vertical height of the chamber. The experimental set-up allowed to resolve sinking rates within the range 0.30 to 8.78 m d^−1^. Cells sinking faster than 8.78 m d^−1^ would have reached the bottom of the chamber before 2 h (first sampling point) and cells sinking slower than 0.30 m d^−1^ would remain in suspension at final sampling time. Sinking cells for any one species and viability status were generally observed to have reached the bottom of the sedimentation chamber at more than one sampling time, indicating that cells within the population exhibited a range of sinking velocities. This observed distribution would be characterized by a modal velocity, i.e. the sedimentation rate most cells exhibited. The distribution of sedimentation rates is reflected in the distribution of cells added to the chamber retrieved at different sampling times.

The modal sedimentation rate for any one species and viability was inferred from the time-series of the cell population retrieved at different sampling times (Fig. S5). We first assigned the modal sedimentation time for a given taxa and viability as corresponding to the time at which the highest cumulative number of cells were retrieved (Fig. S5, e.g. 16 hours in panel B). Once that time was known, we estimated the modal sedimentation rate, which was based on our chamber dimensions, graphically displayed in Fig. S5A, where the minimum and maximum rates constrained by the range that could be resolved by the experimental set up (0.30 to 8.78 m d^−1^) —this was due to the chamber height and the sampling time points. These modal sedimentation rates are conservative (Table S2); as the sedimentation rate for cells retrieved at the sampling yielding the highest fraction of cells (t_x_, e.g. 16 hours in Fig. S5B) would corresponding to cells reaching the bottom of the chamber between times t_x−1_ and t_x_ (e.g. between 6–16 hours, Fig. S5B).

### Statistics

We used non-parametric statistical tests. A two-tailed Mann-Whitney U test was conducted to examine whether significant differences in sedimentation data were observed between living and dead species when all experiment dates and species were pooled. To evaluate whether significant differences in living and dead diatom sedimentation rates were driven by variability among sample dates, a non-parametric one-way ANOVA (Kruskal-Wallis test) was run for both living and dead cells with time. Additionally, a two-tail non-parametric Spearman Rho test was run to examine correlations among sedimentation and diatom net growth rates, along with the trends in the historical data sets between *p*CO_2_, Si(OH)_4_, and NO_3_ + NO_2_. Model-II reduced major axis linear regression analyses were used to compare Si(OH)_4_, and NO_3_ + NO_2_ drawdown for this and previous data sets.

## Supplementary information

Supplementary Information

## Data Availability

The original data can be found in the Figures, Tables, and Supplementary Information or are available upon request. Time-series data at station GF3 in Godthåbsfjord are available through the Greenland Ecosystem Monitoring Database (data.g-e-m.dk). GLODAP data are available as an oceanographic dataset in Ocean Data View (created by R. Schlitzer, odv.awi.de). ICES Oceanographic data are also freely accessible (ocean.ices.dk). Gridded *p*CO_2_ and associated nutrient data are also freely accessible and available through Columbia University (ldeo.columbia.edu/res/pi/CO2/carbondioxide/pages/global_ph.html).
