# Supplementary material for: Silicic acid limitation drives bloom termination and potential carbon sequestration in an Arctic bloom
Source: Sci Rep. 2019 May 31;9:8149. doi: 10.1038/s41598-019-44587-4 (PMC6544819; doi:10.1038/s41598-019-44587-4)
Supplement: Supplementary file 1 — Supplementary Information [file 41598_2019_44587_MOESM1_ESM.docx]

Supplementary Information for

Silicic acid limitation drives bloom termination and potential carbon sequestration in an Arctic bloom

Jeffrey W. Krause^1,2,6,*^, Isabelle K. Schulz^3,6^, Katherine A. Rowe^3^, William Dobbins^1^, Mie H. S. Winding^4^, Mikael K. Sejr^5^, Carlos M. Duarte^3,5^ and Susana Agustí^3^

^1^ Dauphin Island Sea Lab, Dauphin Island, AL, USA

^2^ Department of Marine Sciences, University of South Alabama, Mobile, AL, USA

^3^ Red Sea Research Center, King Abdullah University of Science and Technology, Thuwal, Kingdom of Saudi-Arabia

^4^ Greenland Climate Research Centre, Greenland Institute of Natural Resources, Nuuk, Greenland

^5^ Arctic Research Center (ARC), Aarhus University, Aarhus, Denmark

^6^These authors contributed equally

*Correspondence email: [jkrause@disl.edu](mailto:jkrause@disl.eduU), ORCID: 0000-0003-2479-6229

**This PDF file includes:**

Figs. S1 to S5

Tables S1 to S4

References


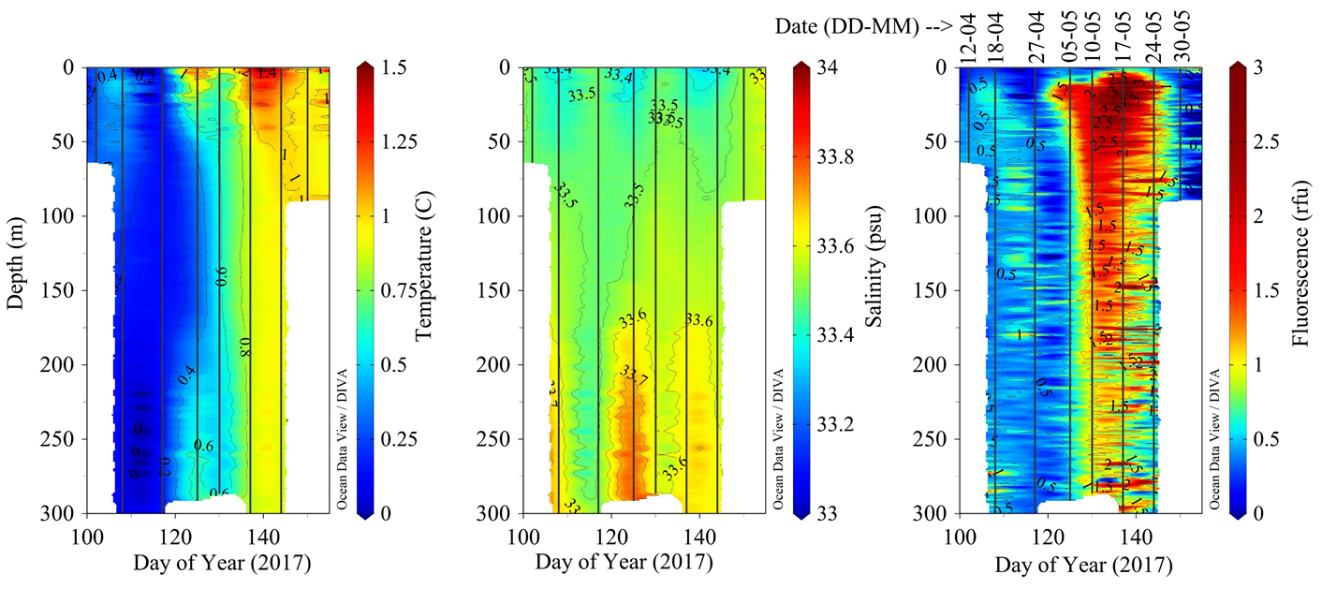
Fig. S1. CTD-derived section plots of temperature, salinity and fluorescence through time at GF3. Data was binned by 1-db pressure using SeaBird processing software.

Fig. S2. Eight-point kinetic curves of silicon uptake as a function of [Si(OH)_4_] (µM) from 18 April through 30 May. Ambient [Si(OH)_4_] (i.e. no addition) is the mean of triplicate samples, the other seven points are single measurements.


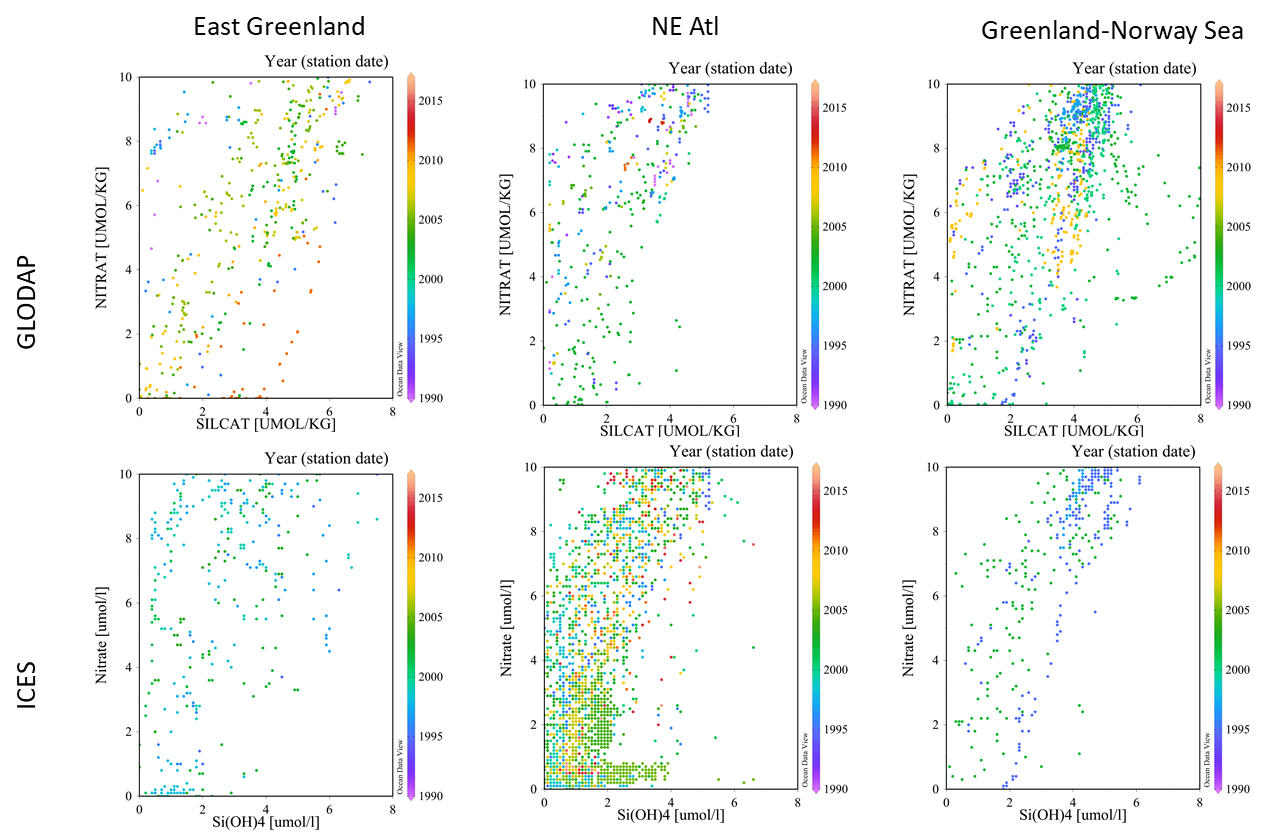


**Fig. S3.** Distribution of nitrate+nitrate vs. silicic acid data in GLODAP and ICES data sets among regions denoted in Fig. 4B. Color scale indicates the year of observation.

**
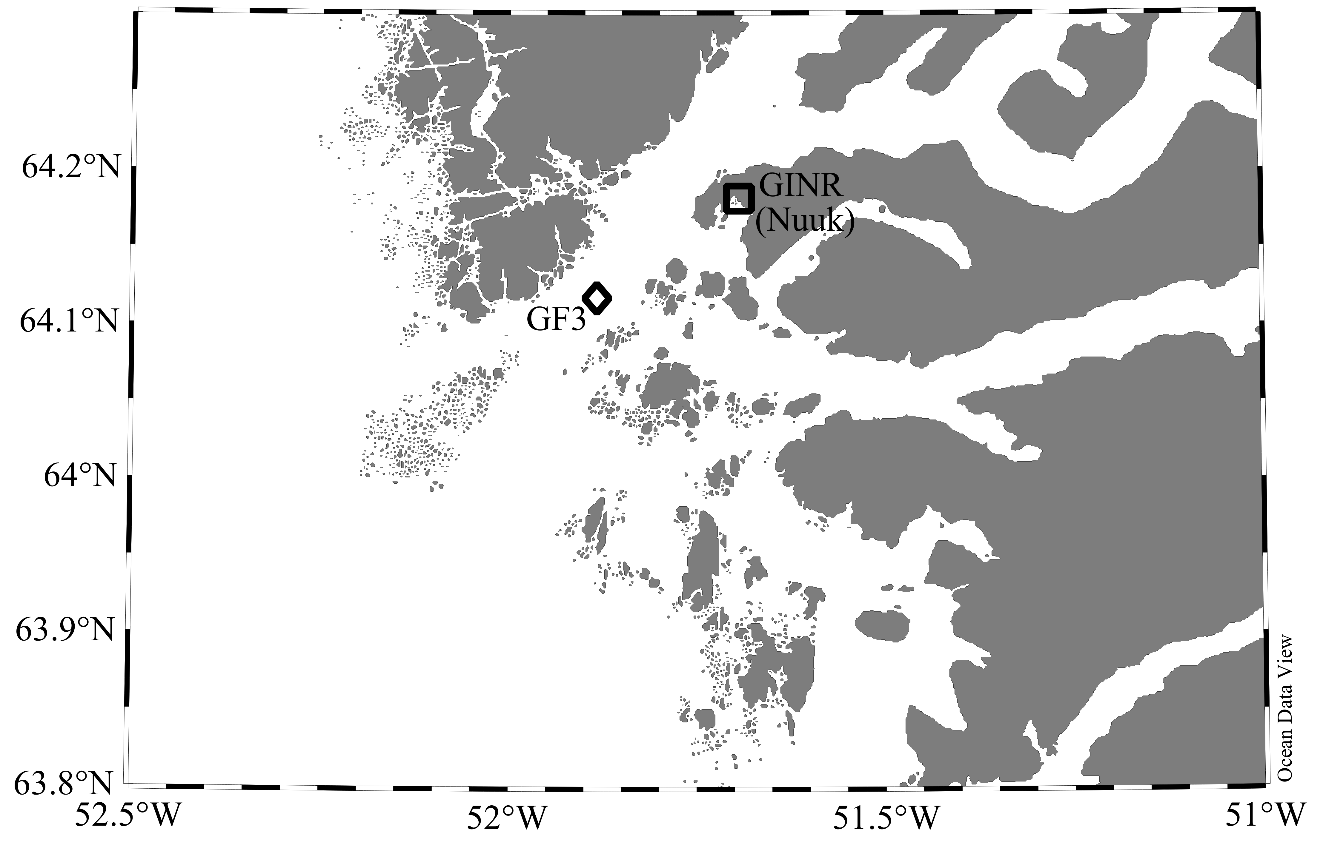
**

**Fig. S4.** Map of study site (GF3) relative to the Greenland Institute of Natural Resources (GINR) located in Nuuk, Greenland. The GF3 site sits near the entrance to Godthåbsfjord. Sampling location relative to the entire AASP is shown on Fig. 4B.

**
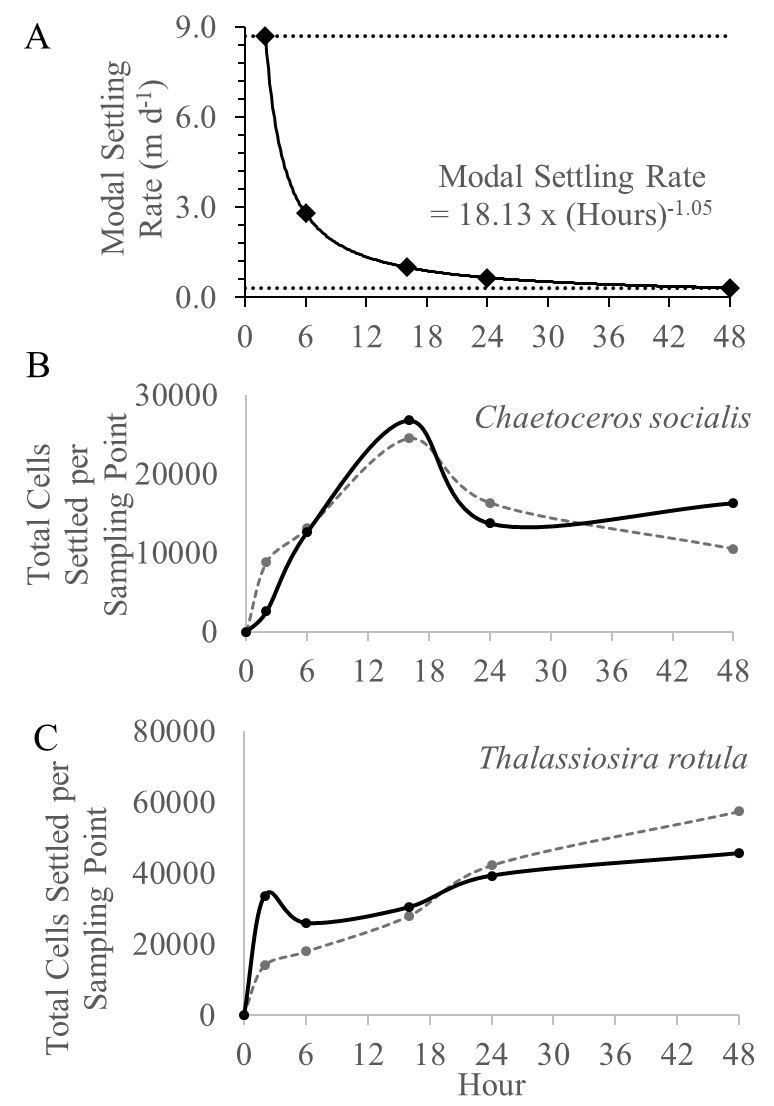
**

**Fig. S5.** A) Settling rate equation, given the dimensions of our apparatus, used to calculate the modal settling rate based on the hour of the observed modal peak. Examples of cells from 10 May (duplicates from different settling columns): B) *C. socialis*, modal peak is 16 hours (rate = 1 m d^-1^), C) *T. rotula*, modal peak at 48 hours (rate = 0.31 m d^-1^).

Table S1. 5-m Niskin bottle data for diatom species abundance (top) and biovolume (bottom) among sampling dates (MM/DD/YY). “0” value denotes species not observed.

| **Species (cells L^-1^)** | 04/12/17 | 04/18/17 | 04/27/17 | 05/05/17 | 05/10/17 | 05/17/17 | 05/24/17 | 05/30/17 |
| --- | --- | --- | --- | --- | --- | --- | --- | --- |
| *Bacterosira bathyomphala* | 0 | 0 | 0 | 14833 | 47000 | 42167 | 29167 | 15250 |
| *Chaetoceros socialis* | 5500 | 5250 | 7250 | 43667 | 74833 | 57500 | 49500 | 22500 |
| *Chaetoceros sp. 2* | 0 | 0 | 1250 | 0 | 0 | 0 | 0 | 0 |
| *Chaetoceros sp. 3* | 750 | 1625 | 750 | 5833 | 0 | 500 | 0 | 0 |
| *Chaetoceros sp. 4* | 500 | 2000 | 1750 | 1833 | 2000 | 1167 | 1333 | 0 |
| *Chaetoceros sp. 5* | 0 | 0 | 0 | 2333 | 0 | 833 | 19167 | 7375 |
| *Cylindrotheca closterium* | 500 | 125 | 250 | 667 | 667 | 0 | 0 | 0 |
| *Eucampia sp.* | 0 | 0 | 0 | 24000 | 3833 | 2167 | 0 | 0 |
| *Navicula sp.* | 625 | 0 | 375 | 1333 | 1667 | 833 | 167 | 0 |
| *Navicula pelagica* | 0 | 0 | 0 | 1000 | 6833 | 3167 | 500 | 1875 |
| *Pleurosigma sp.* | 0 | 0 | 250 | 0 | 0 | 0 | 0 | 0 |
| *Pseudo-nitzschia delicatissima* | 0 | 0 | 0 | 0 | 0 | 0 | 833 | 0 |
| *Rhizosolenia sp.* | 0 | 0 | 0 | 0 | 0 | 0 | 0 | 375 |
| *Thalassiosira sp.(rotula)* | 26750 | 27625 | 78375 | 101500 | 135333 | 101667 | 59667 | 29375 |
| TOTAL | 34625 | 36625 | 90250 | 197000 | 272167 | 210000 | 160333 | 76750 |
| % *Bacterosira* + *Thalassiosira* | 77% | 75% | 87% | 59% | 67% | 68% | 55% | 58% |
|  |  |  |  |  |  |  |  |  |
| **Species (10^6^ µm^3^ L^-1^)** | 04/12/17 | 04/18/17 | 04/27/17 | 05/05/17 | 05/10/17 | 05/17/17 | 05/24/17 | 05/30/17 |
| *Bacterosira bathyomphala* | 0 | 0 | 0 | 143.01 | 453.13 | 406.53 | 281.20 | 147.03 |
| *Chaetoceros socialis* | 1.56 | 1.48 | 2.05 | 12.35 | 21.16 | 16.26 | 14.00 | 6.36 |
| *Chaetoceros sp. 2* | 0 | 0 | 1.25 | 0 | 0 | 0 | 0 | 0 |
| *Chaetoceros sp. 3* | 0.68 | 1.48 | 0.68 | 5.32 | 0 | 0.46 | 0 | 0 |
| *Chaetoceros sp. 4* | 2.76 | 11.05 | 9.67 | 10.13 | 11.05 | 6.45 | 7.37 | 0 |
| *Chaetoceros sp. 5* | 0 | 0 | 0 | 29.23 | 0 | 10.44 | 240.10 | 92.39 |
| *Cylindrotheca closterium* | 0.15 | 0.04 | 0.08 | 0.20 | 0.20 | 0 | 0 | 0 |
| *Eucampia sp.* | 0 | 0 | 0 | 42.22 | 6.74 | 3.81 | 0 | 0 |
| *Navicula sp.* | 0.12 | 0 | 0.07 | 0.26 | 0.33 | 0.16 | 0.03 | 0 |
| *Navicula pelagica* | 0 | 0 | 0 | 0.83 | 5.67 | 2.63 | 0.41 | 1.56 |
| *Pleurosigma sp.* | 0 | 0 | 4.60 | 0 | 0 | 0 | 0 | 0 |
| *Pseudo-nitzschia delicatissima* | 0 | 0 | 0 | 0 | 0 | 0 | 0.07 | 0 |
| *Rhizosolenia sp.* | 0 | 0 | 0 | 0 | 0 | 0 | 0 | 3.75 |
| *Thalassiosira sp.(rotula)* | 211.80 | 218.73 | 620.55 | 803.65 | 1071.54 | 804.97 | 472.43 | 232.58 |
| TOTAL | 217 | 233 | 639 | 1047 | 1570 | 1252 | 1016 | 484 |
| % *Bacterosira* + *Thalassiosira* | 98% | 94% | 97% | 90% | 97% | 97% | 74% | 78% |

Table S2. Modal sedimentation rates among diatom species (live and dead) and date. “nd” denotes species was observed in the 5-m Niskin sample, but was not significantly abundant in the net sample to quantify sedimentation during the experiment.

| **Live Cell Sedimentation (m d^-1^)** | 05/05/17 | 05/10/17 | 05/17/17 | 05/24/17 | 05/30/17 |
| --- | --- | --- | --- | --- | --- |
| *Bacterosira bathyomphala* | 4.49 | 0.53 | 0.36 | 0.60 | 1.81 |
| *Chaetoceros socialis* | 2.33 | 1.03 | 0.63 | 0.50 | 2.74 |
| *Chaetoceros sp. 2* | nd | nd | nd | nd | nd |
| *Chaetoceros sp. 3* | 8.78 | 0.76 | 0.35 | 0.37 | 4.20 |
| *Chaetoceros sp. 4* | 5.12 | 0.30 | 0.84 | 0.71 | nd |
| *Chaetoceros sp. 5* | nd | nd | 0.30 | 2.01 | nd |
| *Cylindrotheca closterium* | 5.12 | nd | nd | nd | nd |
| *Eucampia sp.* | 4.68 | nd | 0.35 | 0.86 | 1.66 |
| *Navicula sp.* | 3.48 | nd | 0.37 | 0.92 | 1.72 |
| *Navicula pelagica* | 4.89 | 0.30 | 0.34 | 0.60 | 2.42 |
| *Pleurosigma sp.* | nd | nd | nd | nd | nd |
| *Pseudo-nitzschia delicatissima* | nd | nd | nd | nd | nd |
| *Rhizosolenia sp.* | nd | nd | nd | nd | nd |
| *Thalassiosira sp.(rotula)* | 2.65 | 0 | 1.68 | 8.78 | 8.78 |
|  |  |  |  |  |  |
| **Dead Cell Sedimentation (m d^-1^)** | 05/05/17 | 05/10/17 | 05/17/17 | 05/24/17 | 05/30/17 |
| *Bacterosira bathyomphala* | 8.78 | 1.82 | 0.80 | 0.30 | 1.80 |
| *Chaetoceros socialis* | 1.42 | 0.62 | 0.92 | 0.37 | 2.58 |
| *Chaetoceros sp. 2* | nd | nd | nd | nd | nd |
| *Chaetoceros sp. 3* | 8.78 | 1.03 | 0.38 | 0.37 | 2.02 |
| *Chaetoceros sp. 4* | 8.78 | 2.20 | 0.98 | 1.83 | nd |
| *Chaetoceros sp. 5* | nd | nd | 0.30 | 1.90 | nd |
| *Cylindrotheca closterium* | 8.78 | nd | nd | nd | nd |
| *Eucampia sp.* | 8.78 | nd | 0.50 | 0.83 | 1.84 |
| *Navicula sp.* | 4.09 | nd | 0.36 | 1.07 | 2.28 |
| *Navicula pelagica* | 8.78 | 1.03 | 0.42 | 0.30 | 2.48 |
| *Pleurosigma sp.* | nd | nd | nd | nd | nd |
| *Pseudo-nitzschia delicatissima* | nd | nd | nd | nd | nd |
| *Rhizosolenia sp.* | nd | nd | nd | nd | nd |
| *Thalassiosira sp.(rotula)* | 8.78 | 2.00 | 0.72 | 4.62 | 0.89 |

Table S3. Calculation of land glacial melt Si delivery using loading reported previously^1^ (i.e. 20 Gmol Si year^-1^). Area calculations estimated using Google Earth shape file (outline of coastal zone) and reading file in Earth Point ([www.earthpoint.us](http://www.earthpoint.us)).

| Greenland Coastal Area (m^2^) | 1.1 x 10^12^ |
| --- | --- |
| Loading (Gmol Si year^-1^) | 20 |
| Loading (mmol Si year^-1^) | 2.0 x 10^13^ |
| *Loading (mmol Si m^-2^ year^-1^) | 17.8 |
| *Assumes loading equally distributed among coastal area |  |
| Loading confined to 90 days (mmol Si m^-2^ d^-1^) | 0.20 |
| If within upper 5 m only (mmol Si m^-3^ d^-1^ or µmol Si L^-1^ d^-1^) | 0.05 |

**Table S4. Average (±Stdev) biogenic silica with and without lithogenic silica correlations for each sampling data and bioassay experiment (triplicate samples).**

| Date (MM/DD/YY) | Experiment Time Point (h) | Treatment | bSi  (µmol Si L^-1^) | bSi  (µmol Si L^-1^), corrected |
| --- | --- | --- | --- | --- |
|  |  |  |  |  |
|  |  |  |  |  |
| 04/18/17 | 0 | - | 0.26 ±0.02 | 0.14 ±0.02 |
|  | 48 | Control | 0.09 ±0.01 | 0.07 ±0.01 |
|  | 48 | Si+ | 0.09 ±0.01 | 0.06 ±0.01 |
| 04/27/17 | 0 | - | 0.60 ±0.01 | 0.38 ±0.01 |
|  | 48 | Control | 0.48 ±0.15 | 0.37 ±0.09 |
|  | 48 | Si+ | 0.65 ±0.32 | 0.51 ±0.24 |
| 05/05/17 | 0 | - | 1.43 ±0.21 | 1.22 ±0.20 |
|  | 48 | Control | 1.90 ±0.07 | 1.71 ±0.07 |
|  | 48 | Si+ | 2.60 ±0.02 | 2.41 ±0.01 |
| 05/10/17 | 0 | - | 1.67 ±0.05 | 1.49 ±0.06 |
|  | 48 | Control | 2.23 ±0.15 | 2.09 ±0.13 |
|  | 48 | Si+ | 2.61 ±0.13 | 2.45 ±0.18 |
| 05/17/17 | 0 | - | 1.60 ±0.19 | 1.46 ±0.18 |
|  | 48 | Control | 2.65 ±0.16 | 2.47 ±0.17 |
|  | 48 | Si+ | 2.72 ±0.02 | 2.54 ±0.02 |
| 05/24/17 | 0 | - | 1.08 ±0.11 | 0.95 ±0.11 |
|  | 48 | Control | 1.50 ±0.11 | 1.37 ±0.11 |
|  | 48 | Si+ | 2.07 ±0.07 | 1.91 ±0.07 |
| 05/30/17 | 0 | - | 1.37 ±0.08 | 1.07 ±0.02 |
|  | 48 | Control | 1.15 ±0.30 | 0.95 ±0.24 |
|  | 48 | Si+ | 1.24 ±0.05 | 1.06 ±0.05 |

**References**

1. Meire, L. *et al*. High export of dissolved silica from the Greenland Ice Sheet. *Geophysical Research Letters* **43**, 9173-9182 (2016).
